# Safety, Feasibility, and Impact of Enalapril on Cardiorespiratory Physiology and Health in Preterm Infants with Systemic Hypertension and Left Ventricular Diastolic Dysfunction

**DOI:** 10.3390/jcm10194519

**Published:** 2021-09-29

**Authors:** Amy H. Stanford, Melanie Reyes, Danielle R. Rios, Regan E. Giesinger, Jennifer G. Jetton, Adrianne R. Bischoff, Patrick J. McNamara

**Affiliations:** 1 Division of Neonatology, Department of Pediatrics, University of Iowa, 200 Hawkins Drive, Iowa City, IA 52242, USA; amy-stanford@uiowa.edu (A.H.S.); melanie-reyeshernandez@uiowa.edu (M.R.); danielle-r-rios@uiowa.edu (D.R.R.); regan-giesinger@uiowa.edu (R.E.G.); adrianne-rahdebischoff@uiowa.edu (A.R.B.); 2 Division of Pediatric Nephrology, Department of Pediatrics, University of Iowa, 200 Hawkins Drive, Iowa City, IA 52242, USA; jennifer-jetton@uiowa.edu; 3 Department of Internal Medicine, University of Iowa, 200 Hawkins Drive, Iowa City, IA 52242, USA

**Keywords:** left ventricular diastolic dysfunction, systemic hypertension, bronchopulmonary dysplasia, angiotensin-converting enzyme inhibitor, enalapril, cardiac lung disease

## Abstract

Neonatal hypertension has been increasingly recognized in premature infants with bronchopulmonary dysplasia (BPD); of note, a sub-population of these infants may have impaired left ventricular (LV) diastolic function, warranting timely treatment to minimize long term repercussions. In this case series, enalapril, an angiotensin-converting enzyme (ACE) inhibitor, was started in neonates with systemic hypertension and echocardiography signs of LV diastolic dysfunction. A total of 11 patients were included with birth weight of 785 ± 239 grams and gestational age of 25.3 (24, 26.1) weeks. Blood pressure improvement was noticed within 2 weeks of treatment. Improvement in LV diastolic function indices were observed with a reduction in Isovolumic Relaxation Time (IVRT) from 63.1 ± 7.2 to 50.9 ± 7.4 msec and improvement in the left atrium size indexed to aorta (LA:Ao) from1.73 (1.43, 1.88) to 1.23 (1.07, 1.29). Neonatal systemic hypertension is often underappreciated in ex-preterm infants and may be associated with important maladaptive cardiac changes with long term implications. It is biologically plausible that identifying and treating LV diastolic dysfunction in neonates with systemic hypertension may have a positive modulator effect on cardiovascular health in childhood and beyond.

## 1. Introduction

Recent evidence suggests that adults born prematurely are at increased risk of heart failure, hypertension, and decreased exercise capacity, which present earlier and more frequently than their term born counterparts [1,2]. Increased survival of premature infants in recent decades has been associated with a growing number of young adults with comorbidities that may have long term repercussions2. Furthermore, survival rates of those born extremely premature (less than 28 weeks) continues to improve, with smaller and younger infants (less than 24 weeks) surviving to adulthood [3,4,5]. Recent evidence suggests that the risk of long-term cardiovascular complications is up to 17 times higher in infants born at the extremes of prematurity (≤28 weeks’ gestation) [6]. In addition, individuals born preterm may have increased left ventricular mass and smaller internal diameters [2].

Neonatal systemic hypertension is increasingly recognized in the subpopulation of infants with bronchopulmonary dysplasia (BPD); early treatment is warranted to minimize the development of end-organ complications [7,8]. Although the exact mechanism for the development of essential neonatal hypertension remains unknown, immature neonates represent a vulnerable population who are at-risk of cardiovascular comorbidities due to immature adaptative responses and exposures related to being born prematurely [9]. Currently there is no consensus regarding the optimal pharmacologic agent to manage neonatal hypertension, and monotherapy has been described to be of limited effectiveness [7,10,11]. Recent evidence from our group suggests that a sub-population of infants with BPD and systemic hypertension may have impaired left ventricular (LV) diastolic function [12]. However, the relationship between the impact on LV function, systemic hypertension, and respiratory morbidity is poorly understood. There is preliminary evidence that Angiotensin Converting Enzyme (ACE) inhibitors may have a positive modulator role on LV diastolic function in neonates [13]. Since July 2020, infants with systemic hypertension and concern for LV diastolic dysfunction at our institution have received treatment with a long-acting ACE inhibitor, enalapril, according to a standardized protocol and strict serial Targeted Neonatal Echocardiography (TnECHO) monitoring. Our primary aim was to characterize the impact of enalapril on LV diastolic function and cardiorespiratory health in high-risk premature infants.

## 2. Materials and Methods

A retrospective cohort study of neonates with systemic hypertension and echocardiography features of LV diastolic dysfunction who received enalapril was conducted. Patients were identified between July 2020 and July 2021 from the Hemodynamics Program echocardiography database at a large quaternary referral center (University of Iowa Stead Family Children’s Hospital). Routine TnECHO screening of preterm infants born at gestational age ≤30 weeks and/or weight ≤1500 g for chronic pulmonary hypertension (cPH) is performed at the earlier of either 8 postnatal weeks or 36 weeks postmenstrual age to facilitate timely detection.

Enalapril Treatment Protocol: Treatment with enalapril is indicated for patients with concurrent systemic hypertension and TnECHO evidence of LV diastolic dysfunction according to a standardized systemic hypertension guideline (Supplementary Figure S1) developed by our Hemodynamics and Pediatric Nephrology teams. Blood pressure (BP) was measured by the oscillometric method in the right arm (pre-ductal) and in one of the calves (post-ductal), concurrent with all TnECHO assessments using appropriately sized cuffs and when the infant was in a calm state. Routine BP measurement was performed every 4–12 h according to level of respiratory support [ventilated infants (every four hours) and non-ventilated patients (every twelve hours)]. The frequency of monitoring was increased if there was intermittent evidence of elevated BP. Systemic hypertension was defined as systolic BP greater than the 95th percentile for postmenstrual age based on published guidelines [11]. LV diastolic dysfunction was diagnosed if there was echocardiography evidence of prolongation of isovolumic relaxation time (IVRT) (>50 msec) [14,15], elevated E/e’ values, and/or a higher peak A-wave (transmitral flow peak velocity during atrial contraction) than peak E-wave (transmitral flow peak velocity of early diastole (E/A < 1)) [14,15,16]. Enalapril was initiated at 0.04 mg/kg daily for three days, after which, if potassium and creatinine levels were stable, the dose was increased to 0.04 mg/kg twice daily. Plasma creatinine and troponin are routinely measured in all patients prior to initiation of enalapril treatment. BP was monitored every eight hours during the first week of enalapril treatment and if any dose adjustments were made. If the BP remained above the 95th percentile after three days of treatment, further adjustments were made until a maximum of 1.2 mg/kg/day was reached. Per our protocol, infants were not discharged until BPs were within goal and a repeat TnECHO with improved LV diastolic function was obtained.

Clinical Data: The following information were collected: (i) neonatal demographics, including birth weight, gestational age, sex, small for gestational age status; (ii) antenatal medications including antenatal steroids, and magnesium sulfate; (iii) maternal risk factors including premature rupture of membranes (PROM), maternal diabetes, and hypertension; (iv) mode of delivery and Apgar scores; (v) common medications in the neonatal period including number of surfactant doses, receipt of inhaled nitric oxide, medical treatment for patent ductus arteriosus (PDA), and steroid exposure; (vi) morbidities such as need for interventional closure of PDA and time of closure, intraventricular hemorrhage, necrotizing enterocolitis (NEC), culture proven sepsis, and duration of invasive ventilation. Data regarding respiratory status [ventilation mode, positive end-expiratory pressure (PEEP) and fraction of inspired oxygen (FiO_2_)] were collected for each infant at the time of each TnECHO evaluation; immediately prior to initiation of enalapril, and then weekly for up to four weeks. Since most patients were on noninvasive respiratory support, a modified respiratory severity score (mRSS) [17] was calculated as PEEP*FiO_2_ for the patients who were on continuous positive airway pressure (CPAP) or non-invasive neurally adjusted ventilatory assist (NIV-NAVA). In patients receiving supplemental oxygen by low flow nasal cannula, the effective FiO_2_ was recorded [18]. Of note, at the University of Iowa, infants with pre-threshold retinopathy of prematurity are treated with supplemental oxygen to target higher oxygen saturation levels in order to reduce the progression to threshold ROP disease [19]. Therefore, oxygen use in our institution is not a reliable marker of severity of BPD. All clinical data were abstracted by a single investigator (MR) who remained blind to the echocardiography data.

Targeted Neonatal Echocardiography Evaluation: All members of the Neonatal Hemodynamics team had completed at least basic TnECHO training and either had completed or were in the process of completing advanced training based on American Society of Echocardiography guidelines for TnECHO evaluation [20]. All screening assessments were performed according to a standardized protocol that includes comprehensive imaging of intracardiac anatomy, biventricular function, outflow tract concordance and integrity, aortic arch anatomy, pulmonary vein location/flow, and transitional shunts [21]. We collected TnECHO data from the assessment performed prior to enalapril initiation and then approximately two weeks after initiation of therapy upon normalization of BP. 

Studies were performed using the Vivid E90 cardiovascular ultrasound system (GE Medical Systems, Milwaukee, WI, USA) with a 6-MHz high-frequency phased-array transducer probe. Standard two-dimensional, M-mode, color Doppler, pulsed wave (PW) Doppler, and continuous wave (CW) Doppler images were obtained. Analyses of left heart volume loading, LV and right ventricle (RV) systolic function, shunt physiology, pulmonary hemodynamics, and cardiac outputs were performed. All echocardiography analyses were performed using a dedicated workstation (EchoPAC version BT10; GE Medical Systems) by a single trained investigator (A.S.), who was blinded to the clinical information to minimize bias. Measurements were performed according to published methodology [21]. Three consecutive cardiac cycles were evaluated and averaged for each measurement to be used in the study. 

Left Heart Evaluation: Blood flow through the mitral valve was assessed from the apical four-chamber view, with a 2- to 3-mm sample volume PW Doppler placed immediately distal to the tips of the valve leaflets. Peak E wave (early diastolic phase), A wave (atrial systolic phase), and their ratio (E/A) were obtained [22]. Isovolumic relaxation time (IVRT) was obtained by placing the sample volume midway between left ventricular outflow and mitral inflow in the apical four-chamber view. Pulmonary vein flow was assessed in the apical four-chamber, with color flow imaging to help sample volume placement, using PW Doppler with the sample volume placed at 1 cm depth into the right (or left) upper pulmonary vein. Left atrium (LA) to aortic ratio (LA:Ao) was obtained in the parasternal long axis via M-mode with the cursor placed at the plane of the aortic valve hinges to include the maximal diameter of the LA and in a plane perpendicular to the aortic wall at the level of the aortic valve. Left ventricular internal diameter, at both end-diastole (LVIDd) and end-systole (LVIDs,) and posterior wall thickness, at both end-systole (LVPWs) and end-diastole (LVPWd), were obtained from the parasternal long axis view with standard M-mode imaging. To assess LV systolic performance, stroke volume (SV) and ejection fraction (EF) via the Simpsons Biplane method were obtained by manual tracing of the LV endocardium at end-systole and end-diastole, from the apical four- and two-chamber views. Fractional shortening (FS) was calculated as follows: (LVIDd-LVIDs)/LVIDd. Left ventricular output (LVO, expressed in mL/min/kg) was calculated by multiplying the aortic cross-sectional area [calculated as: (aortic radius2 × π)] multiplied by velocity time integral (VTI) and heart rate and indexed to weight (kg) [23,24]. To calculate VTI, a PW Doppler sample volume was placed at the level of the aortic valve hinge points, perpendicular to the aortic valve in the apical five-chamber view, with the angle of insonation parallel to the LV outflow tract. The area under the wave form of the aortic systolic beat was traced to obtain the VTI and the heart rate. The annulus of the aortic valve was measured, from the parasternal long axis view, between hinge points with the valve open at the end of ejection. 

LV diastolic function: Conventional (IVRT, E/A, E/e′ ratio) and advanced (peak systolic rate, early diastolic strain rate, and pump function strain rate) indices of diastolic function were evaluated as follows. Tissue Doppler imaging (TDI) velocities were obtained in the apical four- and two-chamber views using a PW Doppler sample volume of 1–2 mm to measure peak systolic (s′), early diastolic (e′) and late diastolic (a′) velocities as well as systolic and diastolic duration. TDI was analyzed from the interventricular septum in the apical four-chamber view and the LV inferior wall from the apical two-chamber view based on enhanced image consistency and straighter angle of insonation. Two-dimensional speckle tracking echocardiography (2D STE) was used to measure longitudinal deformation of the LV in the apical four-chamber view. A frame rate of 80–100 frames/s was used for storage and analysis and only images that were optimized to visualize the myocardial walls were used. The region of interest (ROI) was defined by tracing the endocardial border of the myocardium in end-systole, adjusting the width to match the wall of interest. Tracking was automatic and its acceptability was visually inspected, irrespective of the software’s automatic suggestion, and the appropriate boundaries confirmed. If the tracking was deemed to be suboptimal, the endocardial border was retraced; however, if satisfactory tracking was not achieved within 5 min, the non-tracking segments were excluded from analysis. We accepted global strain values if at least 5 out of 6 segments had acceptable tracking. The period of interest was defined according to the beginning of the QRS complex on the electrocardiogram and the aortic valve closure timing obtained from pulse-waved Doppler imaging of the left ventricular outflow tract. Peak longitudinal strain and strain rates (peak systolic, early diastolic and pump function) were obtained from the from the global tracing. We observed that early diastolic and pump function strain rates were frequently fused and therefore these measurements were not recorded.

Right heart function: Assessment of RV systolic performance included tricuspid annular plane systolic excursion (TAPSE), fractional area change (FAC), and TDI. TAPSE was obtained using M-mode echocardiography with the line of interrogation passing through the lateral aspect of the tricuspid annulus while maintaining vertical alignment with the apex in the apical four-chamber view. From the RV three chamber view, the RV areas at end-diastole and end-systole were calculated by tracing the endocardial borders, including the RV trabeculations within the area. FAC (expressed as a percentage) was calculated using the formula [(RV area at end-diastole − RV area at end-systole)/ RV area at end-diastole]. TDI of the tricuspid annulus was obtained just below the lateral tricuspid annulus in the apical four-chamber [25]. 2D-STE was used to measure longitudinal deformation in the RV three-chamber view utilizing the same procedure outlined above with the period of interest defined according to the beginning of the QRS complex on the electrocardiogram and the pulmonary valve closure timing obtained from PW Doppler imaging of the right ventricular outflow tract on the same view. 

Afterload evaluation: Surrogate indices of pulmonary and systemic vascular resistance and afterload were evaluated [26]. Pulmonary vascular resistance index (PVRi) was evaluated with the ratio of RV ejection time (RVET) to pulmonary artery acceleration time (PAAT) obtained with PW Doppler perpendicular to the pulmonary valve in the parasternal short axis view, with the angle of insonation parallel to the right ventricular outflow tract [27]. RVET was measured as the time from onset to end of systolic flow; PAAT was measured as the time interval between the beginning of systolic flow to its peak velocity [27]. Systemic vascular resistance index was evaluated in a similar way as pulmonary vascular resistance with LV ejection time (LVET) to aortic acceleration time (AoAT) [26]. The systolic time intervals of flow through the aortic valve were measured using PW Doppler from the same view of the LVO. LVET was measured as the time from onset to end of systolic flow. AoAT was defined as the time interval between the beginning of systolic flow to its peak velocity. While LVET: AoAT has been used to assess severity of aortic stenosis in adults^26^, its use in neonates is limited. Left ventricular end-systolic wall stress (ESWS) (g/cm^2^) was calculated using mean blood pressure (MBP) from the right arm oscillometric measurement at the time of TnECHO and M-mode derived indices as per the following formula: (1.35 × MBP × LVIDs)/(4 × LVPWs × (1 + LVPWs/LVIDs)) [28,29]. 

Outcomes: The primary outcome was change in either IVRT, E/A ratio or E/e′. Secondary outcomes included indices of cardiorespiratory stability and echocardiography indices of pulmonary hemodynamics, LV or RV systolic performance. 

Statistical analysis: Descriptive statistics were used for demographics and clinical data. The Shapiro–Wilk test was used to test continuous variables for normality. Mean with standard deviation and median with interquartile range were calculated for data with normal and non-normal distribution, respectively. Comparative evaluation of pre- and post-enalapril clinical and TnECHO variables was performed using paired t-test for normally distributed variables and Wilcoxon Signed Rank test for non-normally distributed variables. Categorical variables were presented as frequencies (%) and compared using the χ^2^ or Fisher’s exact test. Results were considered significant if *p* < 0.01, based on Bonferroni correction for three variables which constituted the primary outcome. Data was analyzed using SPSS version 27 statistical software (IBM, Armonk, NY, USA). No sample size calculation was performed as there are insufficient data regarding the impact of enalapril on LV diastolic function; therefore, for this hypothesis generating study, we used a sample size of convenience.

## 3. Results

Between July 2020 and July 2021 a total of eleven patients were identified who received enalapril therapy for late onset systemic hypertension and LV diastolic dysfunction. All eleven eligible infants were included in this study, of whom three were born less than 24 weeks gestation. Baseline neonatal demographics and indices of illness severity are outlined in Table 1. Birth weight and gestational age were 785 ± 239 grams and 25.3 (24, 26.1) weeks respectively. Nine patients (82%) patients had a hemodynamically significant PDA which required intervention, of whom five (46%) required percutaneous device closure in the cardiac catherization lab. Mean age at receipt of enalapril treatment was 90 ± 20 days. Mean systolic and diastolic arterial pressure parameters at initiation of enalapril were 98 ± 9 mmHg and 56 ± 6 mmHg respectively. Nine infants were receiving non-invasive respiratory support with positive pressure ventilation at time of initiation of enalapril therapy, of whom three were also receiving systemic steroids (hydrocortisone (*n* = 1), dexamethasone (*n* = 1), prednisolone (*n* = 1)) as scheduled tapers for management of bronchopulmonary dysplasia. Additionally, two infants were on nasal canula at time of initiation of enalapril therapy.

As part of the workup for systemic hypertension, renal ultrasound with Doppler was obtained in all patients. All studies were reported as normal, although one patient had diffuse echogenicity that was not attributed to renal intraparenchymal disease. Baseline plasma troponin and creatinine levels were also normal in all cases. Four patients developed systemic hypertension prior to 36 weeks postmenstrual age; therefore, amlodipine treatment was initiated (Table 1). Despite adequate BP control with amlodipine, there was ongoing evidence and/or worsening of LV diastolic function parameters and therefore these infants were transitioned to enalapril.

**Figure 1:**
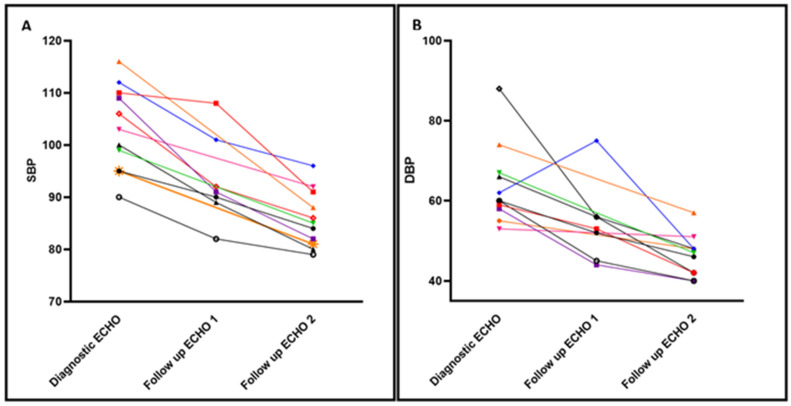
Time-series trends in the Systolic and Diastolic Arterial pressure before/after enalapril. Individual Systolic Blood Pressure (SBP) (Panel **A**) and Diastolic Blood Pressure (DBP) (Panel **B**) trends at three time points. At time of diagnostic echocardiogram (ECHO), and with two follow up ECHOs. ECHO 1: in average 2 weeks after starting enalapril therapy and, ECHO 2: prior to discharge. Each patient is represented in a different color and vignette. 4 patients did not undergo ECHO assessment at 2 weeks after treatment, and only had one follow up ECHO prior to discharge.

Impact of Enalapril on Cardiorespiratory Health: Infants were monitored closely for normalization of their BP (Figure 1). Each individual patient showed improvement in both systolic and diastolic blood pressures after initiating enalapril. An improvement in systolic BP to less than the 95th percentile, within 2 weeks of therapy, was demonstrated for all patients. After starting enalapril, none of the patients had elevation in creatinine, electrolyte derangement, or developed hypotension that required holding or decreasing enalapril dosage. Only one patient required two doses of isradipine for SBP > 110, in the first 72 h of enalapril initiation while titrating up to goal dose. All patients were successfully weaned from respiratory support within 60 days and discharged in the subsequent four to six weeks. Two patients were already receiving oxygen supplementation via low flow nasal cannula when enalapril was started, and one patient, due to severe lung hypoplasia, required reintubation and tracheostomy placement. Nine patients were prescribed additional supplemental oxygen for management of ROP progression, which resulted in a slower wean of respiratory support based on ROP exams. All the patients in our cohort were discharged home on oxygen for management of BPD after failing a room air trial with saturations less than 90%. All patients received steroids during their hospitalization for management of respiratory failure. Three patients were finishing scheduled steroid tapers at time of enalapril initiation. After enalapril treatment, only three patients were prescribed steroids to aid in respiratory management, one of whom had Grade 3 BPD, severe lung hypoplasia and underwent a tracheostomy. Other reasons for steroid treatment after enalapril initiation were stress dosing for surgery in a patient with adrenal insufficiency, and ROP progression despite maintaining respiratory support and oxygen levels (*n* = 2). In our unit, chlorothiazide is used frequently in the management of BPD. After enalapril initiation, chlorothiazide use decreased from 72.7% (*n* = 8) to 45.5% (*n* = 5). In addition, lower dosing required prior to home going (30 to 20 mg/kg/d) with plans to outgrow discharge dose.

**Figure 2:**
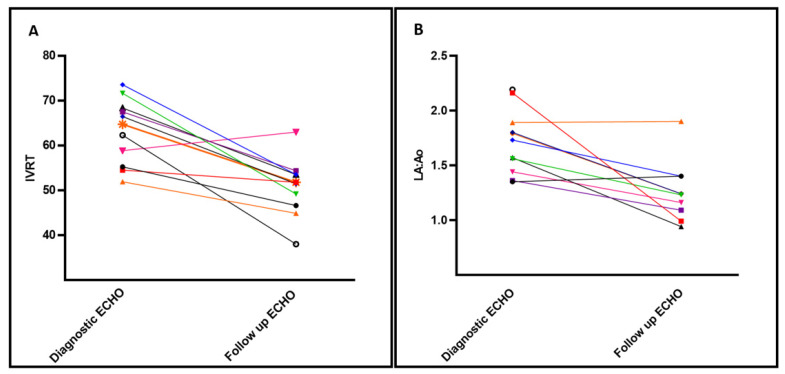
Select echocardiography indices of left heart diastolic function before/after enalapril treatment. Individual changes to Isoloumic Relaxation Time (IVRT) (Panel **A**) and Left Atrium indexed to Aortic size (LA:Ao) (Panel **B**) after treatment with enalapril, baseline shown at time of diagnostic ECHO vs Follow up ECHO prior to discharge. Each patient is represented in a different color and vignette.

Impact of Enalapril on TnECHO indices: Table 2 depicts the changes in TnECHO indices prior to and after initiation of enalapril (mean 10.3 ± 3 days). There was a statistically significant decrease in IVRT and smaller LA:Ao ratio and trend towards reduction in the E/e’ in the inferior wall within two weeks of therapy. In addition, there was a non-significant trend towards decreased LVET: AoAT and ESWS, suggestive of lower systemic vascular resistance. Indices of LV and RV systolic performance remained stable throughout the treatment period. LV diastolic strain rate and pump function strain rates were unable to be analyzed due to several patients with fused early diastolic and pump function waves. Individual patient trend shows improvement in IVRT and La:Ao in all but one patient after enalapril treatment (Figure 2).

## 4. Discussion

In this observational study of infants with systemic hypertension and impaired LV diastolic performance, improvements in BP and echocardiography indices after treatment with enalapril were noted; specifically, a reduction in IVRT which coincided with normalization of left heart volume loading (LA:Ao ratio) and trends towards improvement in LV afterload (ESWS, LVET:AoAT) were noted. Although this is a small cohort study, these data suggest a potentially important relationship between systemic BP and LV diastolic function which is potentially modifiable with ACE inhibitor treatment. In the context of the increasing scientific evidence that adults born prematurely are at increased risk of hypertension, cardiac maldevelopment, and heart failure, our data highlights the importance of prospective characterization of the determinants of cardiac performance.

The immature myocardium contains underdeveloped contractile machinery with disorganized myofibrils, an immature calcium handling system, and inadequately compliant collagen, which predisposes the neonatal heart to diastolic dysfunction and poor tolerance to increased afterload [30]. As preterm infants reach term equivalent gestation, the maturation of the myocardium should occur and inherent LV diastolic dysfunction should improve. It is plausible that in a subset of neonates, this process is disrupted leading to continued LV diastolic dysfunction [31,32]. Untreated, impaired LV compliance and/or impaired relaxation over a prolonged time-period may contribute to cardiac-lung disease [33] with subsequent development of pulmonary venous hypertension and pulmonary edema. Currently, the approach to infants with BPD focuses exclusively on lung-specific therapies, some of which may have unanticipated negative effects. For example, use of selective pulmonary vasodilators in the setting of lung disease due to unrecognized pulmonary venous hypertension, secondary to LV diastolic dysfunction and systemic hypertension, may increase the likelihood and magnitude of pulmonary edema. In addition to myocardial immaturity, certain therapies or disease states may have an additive negative modulator effect. Universal receipt of steroid treatment and high rate of prolonged exposure to hemodynamically significant PDA in our cohort are noteworthy biological considerations. 

Prematurity may disrupt the normal deposition of elastin in the large conduit arteries that typically occurs around term. This interruption in normal development may be further compounded by perinatal interventions such as oxygen and steroids. Survivors of prematurity in an era when generous oxygen use was the standard practice, had increased aortic stiffness [34]. The use of postnatal steroids for hemodynamic support or lung rehabilitation can improve the survival of preterm infants, but their impact on fetal, neonatal, and long-term cardiovascular development is unclear [35]. When given in utero, endogenous glucocorticoids promote morphological remodeling in fetal cardiomyocytes with cardiomyocyte proliferation dictating the morphological development of the heart. Postnatal steroid use is associated with altered LV geometry, hypertrophic cardiomyopathy, and systemic hypertension in neonates [36]. Furthermore, aortic stiffness has been observed in young adults exposed to antenatal steroids [37] and in a lamb model with decreased elastin, smooth muscle deposition and disrupted elastin deposition [38]. Steroid exposure in utero and after birth may additionally predispose infants to further LV diastolic dysfunction due to inherent changes in cardiac morphology with thicker and stiffer hearts. The likelihood of need for steroid therapy is greatest in infants born at the limits of viability, as in our cohort, who represent the population at greatest risk of cardiac maldevelopment. 

Persistent exposure to a high-volume PDA shunt can have serious hemodynamic consequences including both pulmonary over circulation and systemic hypoperfusion, which may have an additional negative modulator effect on LV diastolic function through coronary hypoperfusion [39]. Additionally, with prolonged exposure to high volume shunts, renal hypoperfusion may occur resulting in upregulation of the renin-angiotensin-aldosterone system (RAAS) and consequently systemic hypertension. Furthermore, prematurity independently may cause disruption of RAAS [40] leading to increased sympathetic nerve activity and increased plasma catecholamines, which may lead to vasoconstrictor effects of catecholamines causing hypertension [41,42]. The involvement of RAAS in the development of abnormalities in the cardiovascular system and lung has recently been identified [43,44]. Renin cleaves angiotensinogen into angiotensin I (Ang I) which is then converted into angiotensin II (Ang II) by ACE. Angiotensin II exerts numerous effects on arterial vasculature including causing endothelial dysfunction with smooth muscle hypertrophy and proliferation of myocytes, fibroblastic proliferation, myocardial fibrosis, and vasoconstriction [13]. Disrupted elastin deposition of the aorta or upregulation of RAAS resulting in increased afterload, particularly when paired with a relatively stiff ventricular system, increases stroke work and myocardial oxygen consumption, with decreased cardiac efficiency. These factors may influence not only increased respiratory morbidity in infancy due to cardiac-lung disease [33] but also cardiac development and increased risk for cardiovascular disease and decreased tolerance to exercise later in life [45]. Development of cardiac alterations in adulthood, including fibrosis and hypertrophy have been linked to activation of the RAAS, in particular due to angiotensin II [1,46,47].

Targeted therapies which act on the RAAS system may offer a novel approach to infants with cardiac-lung disease. Inhibition of ACE rather than angiotensin receptor blockers [48] has been shown to improve endothelial function, alter vascular remodeling with decreased cardiomyocyte hypertrophy (with inhibition of interstitial fibrosis leading to a reduction in ventricular cavity size), and additionally increase the production of prostaglandin E2, resulting in vasodilation and subsequent decreases in peripheral vascular resistance and therefore decreases in afterload [13,46,49,50,51,52,53]. Enalapril is an ACE inhibitor which is commonly used as first line therapy for its afterload reduction effects in children or adolescents with heart failure, LV systolic dysfunction, or systemic hypertension [54,55,56,57]. Emerging literature supports its potential use as a therapeutic agent in BPD [13,33]. Enalapril, with its anti-remodeling effects, may be an ideal drug to treat systemic hypertension in the setting of LV diastolic dysfunction in ex-preterm neonates.

Limitations: There are several important limitations in this study particularly related to its retrospective nature and small sample size. First, some infants with systemic hypertension and LV diastolic dysfunction were treated initially with amlodipine as enalapril is not started before 36 weeks PMA due to the concern for potential nephrotoxicity during nephrogenesis. Once infants are greater than 36 weeks PMA, with signs of LV diastolic dysfunction, they are transitioned to enalapril as nephrogenesis is nearing completion [58]. It is interesting to note that all four infants previously treated with amlodipine still had impaired LV diastolic function despite improvement in BP, which may suggest that ACE inhibitors may be a superior treatment in the setting of LV diastolic dysfunction. Second, the follow-up of our patients was not completely standardized. While our protocol is to reassess the patients one week after they are normotensive (typically 2 weeks after initiation of enalapril), in some cases infants were reassessed sooner at the clinical team’s request, most often as the patient was approaching discharge. Third, our data explores enalapril use over a short time-period which either may not have captured the true magnitude of impact on LV diastolic function with sustained blood pressure control over a more prolonged period or may be too early to identify adverse effects of treatment. Fourth, this population had additional changes in respiratory management based on ROP. As previously mentioned, supplemental oxygen is used to target higher oxygen saturation levels for infants with pre-threshold ROP in order to reduce disease progression. Furthermore, when ROP is progressing despite oxygen therapy, additional respiratory management strategies are used, such as increased PEEP and increased steroid therapies to optimize respiratory health and oxygen delivery. These practices bias against true characterization of the impact of modulation of LV diastolic dysfunction on respiratory health. Fifth, we used e′ values from the apical 4-Chamber view at the interventricular septum and the apical 2-chamber view for the LV inferior wall based on enhanced image consistency and optimal angle of insonation. The e′ value for the septum, however, is likely influenced by RV function thus potentially making its value less representative of overall LV diastolic function. In addition, LV diastolic function may not be uniformly impacted across the different walls of the LV. Lastly, due to such a small sample size, we did not perform inter or intra rater reliability which limits the generalizability of our data. 

## 5. Conclusions

In summary, enalapril treatment in infants with systemic hypertension and LV diastolic dysfunction led to interval improvements in blood pressure, left heart volume loading, LV diastolic function, and afterload. These findings are important as systemic hypertension is often underappreciated in ex-preterm infants and may be associated with maladaptive cardiac changes including LV diastolic dysfunction. Reports that adult survivors of prematurity are at increased risk for cardiovascular disease, heart failure, ischemic heart disease, cardiometabolic impairment, and systemic hypertension in early adulthood lends additional credence to the findings [6,35,59,60,61,62]. As individuals progress to adulthood, reductions in diastolic function have diagnostic, therapeutic, and prognostic value [63]. Enhanced mechanistic understanding of myocardial energetics, efficiency, and the relationship between LV diastolic performance and systemic afterload are needed. Although our data are preliminary, and conclusive endorsement of therapeutic efficacy and/or safety should not be implied, it is biologically plausible, that identifying and then treating LV diastolic dysfunction in neonates with systemic hypertension may have a positive modulator effect on cardiovascular health in childhood and beyond.

## Figures and Tables

**Table 1:** Demographics and baseline illness severity in patients who received enalapril therapy.

Demographics and Baseline Illness Severity	*n* = 11
Birth weight (grams)	785 ± 239
Gestational Age (weeks)	25.3 (24, 26.1)
Male sex	4 (36.4)
Small for gestational age	3 (27.3)
Antenatal steroids	8 (72.7)
Antenatal MgSO4	7 (63.6)
PPROM	
<18 h	7 (63.6)
18 h–7 days	0 (0)
>7 days	4 (36.4)
C-section delivery	8 (72.7)
Maternal hypertension	2 (18.2)
Maternal diabetes	1 (9.1)
5-minute APGAR score	6.1 ± 1.9
Surfactant replacement therapy	10 (90.9)
Number of doses of surfactant	1 (1, 2.2)
iNO in the transitional period (1st week of life)	1 (9.1)
Treatment for PDA	
No treatment	2 (18.2)
Medical treatment	4 (36.4)
Interventional closure	5 (45.5)
Doses of acetaminophen	18.1 ± 16.8
Doses of indomethacin	3.9 ± 4.1
Age at definitive PDA closure (days)	31 ± 24.5
Status pre-enalapril	
Age enalapril started (days)	90 ± 20
Right arm Systolic Blood Pressure (mmHg)	98 ± 9
Right arm Diastolic Blood Pressure (mmHg)	56 ± 6
Leg Systolic Blood Pressure (mmHg)	96 ± 9
Leg Diastolic Blood Pressure (mmHg)	55 ± 12
Amlodipine therapy prior to Enalapril (*n*)	4
Dose (mg/kg)	0.1
Length of treatment (days)	20.75 (13, 35)
Isradipine use for SBP > 110 on Enalapril (*n*)	1
Total dose (mg/kg)	0.1
Ventilation mode:	
Invasive MV	0 (0)
Non-invasive respiratory support	9 (81.8)
Nasal cannula	2 (18.2)
PEEP (cmH_2_O) (*n* = 9)	8 (6.5, 12)
Fraction of inspired oxygen (%)	41.2 ± 12
Modified respiratory severity score (*n* = 9)	3.9 ± 1.7
Neonatal morbidities	
Intraventricular hemorrhage	
None	5 (45.5)
Grade I/II	4 (36.4)
Grade III/IV	2 (18.2)
NEC	1 (9.1)
Culture proven sepsis	5 (45.5)
Duration of invasive ventilation (days)	42.6 ± 33
Duration of non-invasive ventilation (days)	59 (52, 81)
Duration of iNO (total during hospitalization) (days)	0 (0, 0)
Medications at the time of enalapril initiation	
Diuretics	8 (72.7)
Systemic steroids *	3 (27.3)
Pulmonary vasodilators	0 (0)
Creatinine baseline (mg/dL)	0.2 (0.1, 0.3)
Troponin T baseline (ng/mL)	0.03 (0.02, 0.03)

MV: mechanical ventilation; HFJV: high frequency jet ventilation; PDA: patent ductus arteriosus, PPROM: preterm premature rupture of membranes, iNO: inhaled nitric oxide, SBP: systolic blood pressure, PEEP: positive end expiratory pressure, NEC: necrotizing enterocolitis. * At time of enalapril initiation, three patients were undergoing steroid tapers for pulmonary management, one with hydrocortisone, one with dexamethasone and one with prednisolone.

**Table 2:** Targeted Neonatal ECHO indices pre and post enalapril therapy in assessment prior to discharge.

	Pre-Enalapril	Post-Enalapril	*p*
Primary outcomes:			
LV diastolic function			
Mitral E/A	0.95 ± 0.2	1 ± 0.1	NS
IVRT (msec)	63.1 ± 7.2	50.9 ± 7.4	0.002
E/e′ septum	13.9 ± 7.6	13.9 ± 5.7	NS
E/e′ inferior	15.1 (8.1, 17)	10.2 (8.2, 17.8)	NS
Secondary outcomes:			
LV diastolic function			
Septum e′ (cm/s)	7.8 ± 3.3	6.9 ± 2.4	
Septum a′ (cm/s)	7.7 ± 3.13	6.8 ± 2	
Systolic: diastolic ratio (septum)	1.26 (1.16, 1.4)	1.25 (1.1, 1.44)	
Inferior e′ (cm/s)	8.7 ± 3.6	7.6 ± 2.6	
Inferior a′ (cm/s)	9.1 ± 2.5	9 ± 3	
Systolic: diastolic ratio (inferior)	1.29 ± 0.24	1.22 ± 0.21	
Left Heart Volume Loading/Systolic Performance			
Pulmonary vein S (cm/s)	50.9 ± 9	55.5 ± 8.6	
Pulmonary vein D (cm/s)	42.6 ± 6.9	43.5 ± 9	
Pulmonary S:D ratio	1.21 ± 0.23	1.31 ± 0.26	
Mitral inflow E (cm/s)	89.6 ± 17.5	87 ± 15.6	
Mitral inflow A (cm/s)	94.6 ± 11.5	87.6 ± 14.1	
LA:Ao	1.73 (1.43, 1.88)	1.23 (1.07, 1.29)	
LVIDd (mm)	1.9 ± 0.2	1.9 ± 0.1	
LVIDs (mm)	1.3 ± 0.3	1.3 ± 0.1	
LVPWD systole (mm)	0.63 ± 0.09	0.68 ± 0.13	
LVPWD diastole (mm)	0.42 ± 0.11	0.46 ± 0.09	
LVO (mL/min/kg)	207 ± 25	192 ± 27	
FS (%)	31.7 ± 12.9	32.4 ± 3.2	
EF (Simpsons Biplane) (%)	66.9 ± 4.9	68.7 ± 4.5	
SV (Simpsons Biplane) (mL/kg)	1.5 ± 0.29	1.43 ± 0.33	
Septum s′ (cm/s)	5.7 ± 1.3	4.8 ± 0.8	
Inferior s′ (cm/s)	5.8 ± 1.2	6 ± 1.7	
Peak longitudinal strain (LV 4Ch) (%)	−16.3 ± 3.2	−15.6 ± 2.8	
Peak systolic strain rate (1/s)	−1.36 ± 0.15	−1.37 ± 0.23	
RV systolic function			
TAPSE (mm)	11.3 ± 1.3	11.5 ± 1.7	
FAC (%)	47.8 ± 8.1	48.5 ± 11	
RV s’ (cm/s)	8.5 ± 0.77	7.7 ± 1	
Peak longitudinal strain (RV 3Ch) (%)	−21.8 ± 1.9	−23.3 ± 4.9	
Vascular resistance			
LV ejection time (msec)	190 ± 24.1	195.3 ± 11.1	
Aortic acceleration time (msec)	38.1 ± 8.1	42.3 ± 7.9	
LVET:AoAT	5.1 ± 1.2	4.7 ± 0.8	
RV ejection time (msec)	196.6 ± 16.5	202.8 ± 16.6	
PA acceleration time (msec)	48.4 ± 8.5	54.2 ± 14.7	
RVET:PAAT	4.1 ± 0.8	3.9 ± 0.8	
LV exposed vascular resistance (dynes/s/cm^5^)	0.31 ± 0.05	0.34 ± 0.08	
ESWS (g/cm^2^)	83.7 ± 22.6	70.8 ± 21.4	

IVRT: isovolumic relaxation time, LA:Ao left atrium size indexed to aorta, LV: left ventricle, LVIDd: Left ventricular internal diameter in diastole, LVIDs: Left ventricular internal diameter in systole. LVPWD Left ventricular posterior wall end-diastole, LVOT: Left ventricular outflow tract, VTI: Ventricular time interval. LVO: Left ventricular output, FS: Fractional shortening, EF: Ejection fraction, SV: systolic volume. RV: Right ventricle, TAPSE: Tricuspid annular plane systolic excursion, FAC: Fractional area change, LVET:AoAT: Left ventricular ejection time indexed to Aortic acceleration time, PA: Pulmonary Artery, RVET:PAAT: RV ejection time indexed to Pulmonic Artery acceleration time, ESWS: End-systolic wall stress, TDI: Tissue doppler index. NS: not significant.

## Supplementary Materials

The following are available online at https://www.mdpi.com/article/10.3390/jcm10194519/s1, Figure S1: Approach to Hemodynamic Care of Premature Infants with Later Systemic Hypertension.
