# Supplementary material for: Safety, Feasibility, and Impact of Enalapril on Cardiorespiratory Physiology and Health in Preterm Infants with Systemic Hypertension and Left Ventricular Diastolic Dysfunction
Source: J Clin Med. 2021 Sep 29;10(19):4519. doi: 10.3390/jcm10194519 (PMC8509219; doi:10.3390/jcm10194519)

Supplementary Figure S1: Approach to Hemodynamic Care of Premature Infants with Later Systemic Hypertension

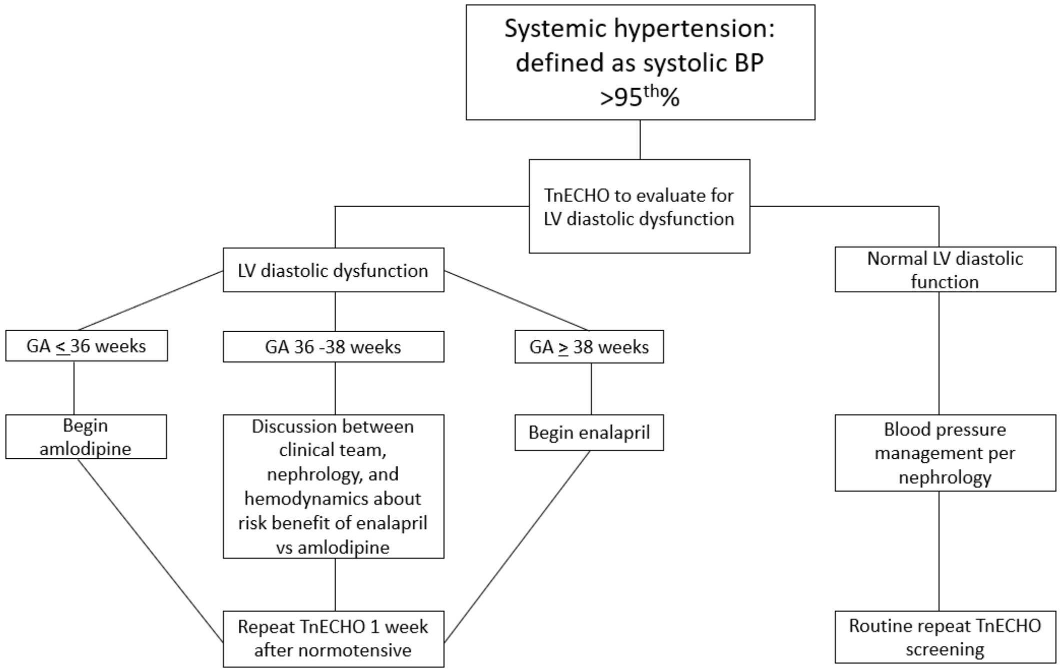

Supplement: Supplementary file 1 [file jcm-10-04519-s001.zip › jcm-1346749-supplementary.pdf]
